# Putative Immunological Functions of Inducible Skin-Associated Lymphoid Tissue in the Context of Mucosa-Associated Lymphoid Tissue

**DOI:** 10.3389/fimmu.2021.733484

**Published:** 2021-08-26

**Authors:** Toshiaki Kogame, Kenji Kabashima, Gyohei Egawa

**Affiliations:** Department of Dermatology, Kyoto University Graduate School of Medicine, Kyoto, Japan

**Keywords:** inducible skin-associated lymphoid tissue, iSALT, MALT, tertiary lymphoid structure (TLS), acquired immunity

## Abstract

Acquired immunity is orchestrated in various lymphoid organs, including bone marrow, thymus, spleen, and lymph nodes in humans. However, mucosa-associated lymphoid tissue (MALT) is evolutionally known to be emerged in the oldest vertebrates as an immunological tissue for acquired immunity, much earlier than the advent of lymph nodes which appeared in endotherms. Furthermore, the lymphocytes which developed in MALT are known to circulate within the limited anatomical areas. Thus, MALT is comprehended as not the structure but the immune network dedicated to local immunity. As for the skin, skin-associated lymphoid tissue (SALT) was previously postulated; however, its existence has not been proven. Our group recently showed that aggregations of dendritic cells, M2 macrophages, and high endothelial venules (HEVs) are essential components to activate effector T cells in the murine contact hypersensitivity model and termed it as inducible SALT (iSALT) since it was a transient entity that serves for acquired immunity of the skin. Furthermore, in various human skin diseases, we reported that the ectopic formation of lymphoid follicles that immunohistochemically analogous to MALT and regarded them as human counterparts of iSALT. These data raised the possibility that SALT can exist as an inducible form, namely iSALT, which shares the biological significance of MALT. In this article, we revisit the evolution of immunological organs and the related components among vertebrates to discuss the conserved functions of MALT. Furthermore, we also discuss the putative characteristics and functions of iSALT in the context of the MALT concept.

## Introduction

The conceptual framework of skin-associated lymphoid tissue (SALT) was proposed by Streilein as early as 1978 (1). It has shed light on the lymphoid properties of the skin for activation of skin-oriented lymphocytes (2, 3). The concept of SALT was postulated based on the investigation of mucosa-associated lymphoid tissue (MALT), which consists of non-encapsulated lymphoid follicles and M cells and serves the purpose of monitoring luminal antigens in the areas covered by mucosa (4). Affinity maturation and class switch recombination of antibodies are conducted in MALT and contribute to local immunity at the mucosal surface *via* IgA production (5).

From an evolutionary point of view, the lamprey, which is the most ancient vertebrate, appearing approximately 500 million years ago (6, 7), lacks lymph nodes and the spleen but contains MALT (8). Although the spleen appeared in cartilaginous fish such as sharks, lymph nodes did not exist in vertebrates until birds developed a functional counterpart of the lymph node, called the bursa of Fabricius (9–11). Thus, the evolutional conservation of MALT suggests the possibility of its involvement with immunity among vertebrates for millions of years. As its name suggests, MALT is not involved in skin immunity. However, it is assumed that the skin, the largest organ of the body serving as the barrier to the outside world, should have its own immune system dedicated to local immunity (12). However, the presence of SALT is still disputed (13).

Dermatological textbooks describe skin diseases accompanied by lymphoid follicles, including lymphocytoma cutis, cutaneous lymphoid hyperplasia, and pseudolymphoma; however, the biological significance of the lymphoid follicles in these diseases is unknown (14). Recently, our group reported that a complex composed of dendritic cells (DCs) and M2 macrophages in the vicinity of blood vessels is essential for activating T cells in a mouse model of contact hypersensitivity (CHS), which we termed inducible SALT (iSALT). Subsequently, we reported that reactive lymphoid follicles in human skin diseases harbor a staining pattern similar to and a structure analogous to those of lymphoid follicles in lymph nodes. These findings led us to hypothesize that lymphoid follicles in the skin are the human counterpart of iSALT.

In this review, we introduce the definition of MALT and discuss the similarities and differences among MALT, iSALT, and related immune structures. We also discuss the evolutionary aspect of lymphoid organs and subsequently introduce the current progress in iSALT research.

## Definitions of MALT and Tertiary Lymphoid Structures (TLSs)

In humans and mice, the thymus and bone marrow are primary lymphoid organs that produce T and B lymphocytes, respectively, while the lymph node is a secondary lymphoid organ (SLOs) in which B and T cells proliferate in response to antigen-induced acquired immunity (15). The formation of SLOs is a genetically preprogrammed process occurring during embryogenesis. In contrast, the lymphoid tissues that form ectopically after birth, termed TLSs, lack encapsulating membranes (16).

**Figure 1:**
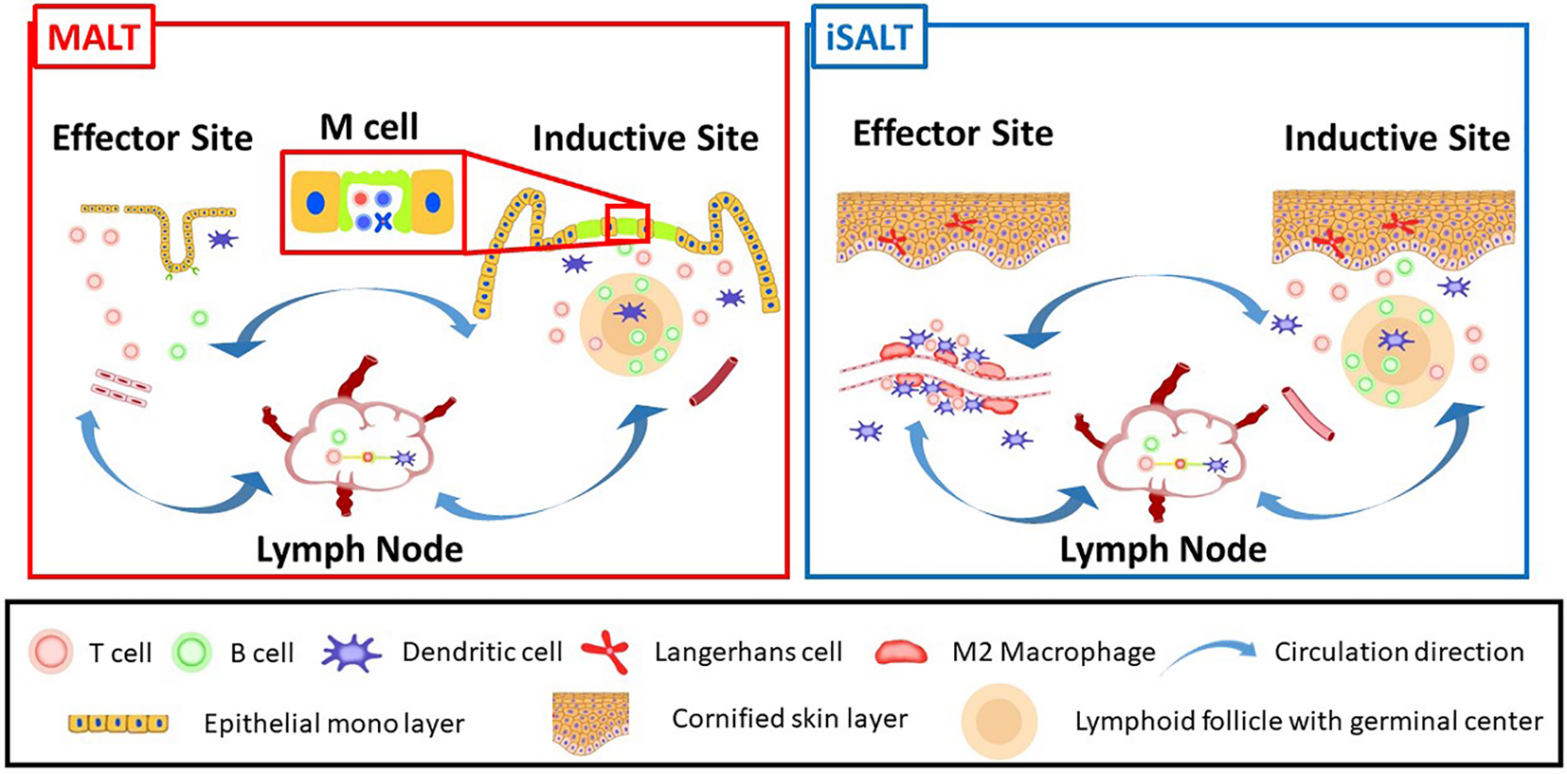
A schematic model of iSALT corresponding to MALT concept: MALT (left) and iSALT (right) are consist of inductive site, effector site, and regional lymph nodes. The inductive site of MALT harbors lymphoid follicles termed as MALT structures which play pivotal roles to induce acquired immunity. M cells locate in the monolayer termed follicle-associated epithelium above MALT structures. Transient induction of lymphoid follicles immunohistochemically consistent with MALT structure is observed in the dermis beneath the cornified epidermis in some human skin diseases, which correspond to inductive sites of iSALT although it is unknown if M cells co-localize. Effector site is an anatomically distant site which the differentiated B and T lymphocytes migrate to, from inductive site or regional lymph nodes. Thus, the effects of immune memory are exerted upon the antigens in effector site. Effector site of MALT is well-investigated for B cell lineage function such as IgA delivery to outside of epithelial monolayer through transcytosis. On the other hand, the phenomena observed in the mouse CHS model suggest the effector site of iSALT where CD8+ T cells, M2 macrophages, and dendritic cells adjacent to blood vessels are essential to activate effector T cells. Langerhans cells are also known as a regulator of inflammation. Lymphocytes differentiated in iSALT are considered to circulate within a limited area of anatomical sites, namely the skin (Circulation directions are indicated by arrow in the figure).

The term MALT was originally coined to emphasize its functions in acquired immunity contributing to local immunity in the mucosa (17). Seminal experiments evaluating the secretory IgA system validated the concept of MALT, which consists of inductive and effector sites (**Figure 1**) (18). The inductive site harbors a lymphoid follicle analogous to lymph nodes and induces lymphocyte differentiation triggered by antigen recognition (**Figure 1**, right side). The effecter site is the destination for developed lymphocytes where they migrate and exert immunological functions (**Figure 1**, left side). It was also demonstrated that the lymphocytes that develop in MALT circulate between the inductive and effector sites *via* regional lymph nodes (**Figure 1**, middle) in anatomically limited areas (19). MALT is divided into gut-associated lymphoid tissue (GALT), nasopharynx/nasal-associated lymphoid tissue (NALT), and bronchus-associated lymphoid tissue (BALT) according to the anatomical site affected (4, 20, 21). Thus, the term MALT is used to describe the local immunity unit of specific anatomical areas. Among MALTs, Peyer’s patch and Waldeyer’s ring are formed prenatally and thus are SLOs, while the remaining MALTs are TLSs. Although there is no clear definition of the functions of TLSs, these structures are expected to be not only morphologically but also functionally analogous to MALT. Thus, the definition of TLSs is based on functional validation of their role in local immunity (4).

On the other hand, in histology-based studies, the term TLS has been used conventionally to encompass other meanings and descriptions, such as ectopically organized structures with segregation of B- and T-cell zones. This structure may provide a site for efficient interactions between lymphocytes and antigen (22). In addition, immunohistochemical analysis showed that the lymphoid follicles in TLSs harbor the same components as those in the lymph node, including plasma cells that differentiate from B cells *via* acquired immunity responses (23). Those histopathological findings suggest that the lymphoid follicles serve as TLSs that are functionally capable of participating in acquired immunity. Therefore, well-organized lymphoid follicles with histopathological findings analogous to those of lymph nodes are generally accepted as TLSs in histology-based research (23–25). In other words, the definition of TLS in histology-based studies does not necessarily require functional validation.

Our group recently reported that lymphoid follicles in the skin exhibit immunohistochemical patterns analogous to those of lymph nodes, and therefore we defined these follicles as iSALT in humans (26–30). Similarly, many lymphoid follicles that fulfill the definition of TLS in histology-based studies have been identified in the salivary duct, in lachrymal drainage, in the Eustachian tube, and in the larynx (31). Nonetheless, according to the strict definition of MALT, these follicles are regarded as lymphoid follicles with the potential to be TLS but not *bona fide* TLS. This discrepancy in the definition of TLS highlights the fundamental question of whether all lymphoid follicles histopathologically analogous to lymph nodes possess local immune function, which is characterized by immune cells circulating between inductive and effector sites. Research to address this question will also determine whether the concept of SALT postulated by Streilein exists. Hereafter, for simplicity, the term TLS or iSALT follows the histology-based definition.

## Components of TLSs and MALT Structures

As mentioned, the concept of MALT describes two anatomically different sites: the inductive and effector sites (**Figure 1**). The inductive site is characterized by distinct lymphoid follicles referred to as MALT structures (**Figure 1**, right side) (4). TLSs are considered MALT structures in various tissues and, therefore, share the same immune structures (4). TLS and MALT structures harbor CD19+ CD20+ B-cell zones located in the center of lymphoid follicles. T cells surround the B-cell zone and form the T-cell zone, which contains high endothelial venules (HEVs); HEVs recruit naïve T and B cells. Follicular DCs (FDCs) expressing the B-cell chemoattractant CXCL13 are located in germinal centers. Germinal center B cells express activation-induced cytidine deaminase, which plays crucial roles in mediating the affinity maturation and class-switch recombination of antibodies. DC-LAMP+ DCs and CD68+ macrophages are scattered over the lymphoid follicles. CD138+ plasma cells with polyclonal IgG expression surround the lymphoid follicles (23). Histological evidence of these components suggests that MALT structures play a role in acquired immunity, similar to lymph nodes (24, 25).

The inductive site harbors MALT structures and M cells in the epithelium of the lumen of the digestive tract. M cells are specialized epithelial cells that capture antigens for the purpose of transporting them across the epithelial membrane (known as transcytosis), rather than phagocytosis (32). B cells and DCs reside in the basolateral pockets of M cells, typically in Peyer’s patch. A triad of M cell, B cells, and DCs is considered to serve for mucosal immune surveillance. Previous studies revealed two types of M cells: constitutive and inducible types (33). Inducible M cells were discovered in inducible BALT in mice. Thus, it would be of great interest if the inductive sites of all TLSs share the same structure: lymphoid follicles and M cells on the follicle-associated epithelium.

## Evolution of Lymphoid Tissue

**Figure 2:**
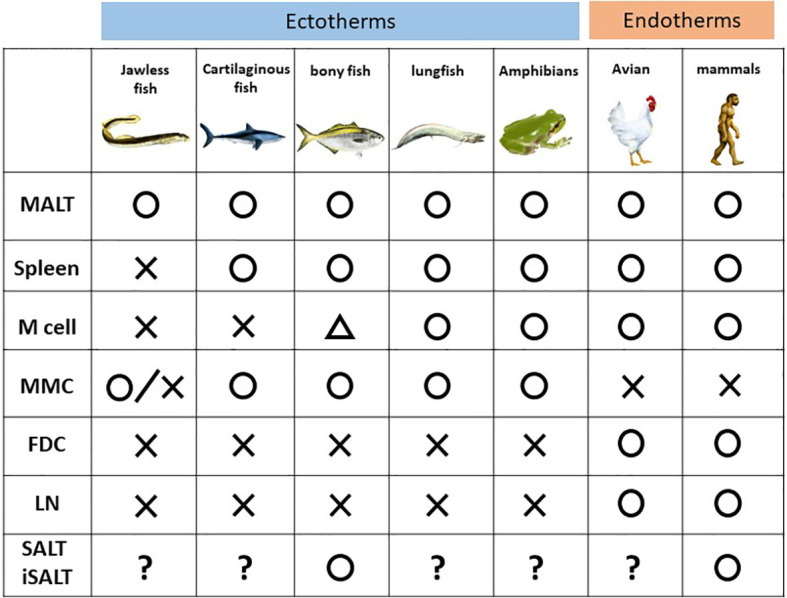
Phylogenic comparison of immune components among the vertebrates. MALT, mucosa-associated lymphoid cells; MMC, melanomacrophage center; FDC, follicular dendritic cells; LN, lymph node and its counterpart; SALT, skin-associated lymphoid cells; iSALT, inducible SALT.

In this section, classes of vertebrates, including jawless fish, cartilaginous fish, bony, amphibians, avian, and mammals, are discussed to clarify the concept of SALT (**Figure 2**).

Among vertebrates, jawless fish such as lamprey and hagfish, a group split off from the rest of other vertebrates 500 million years ago (6), harbor different types of lymphocytes, akin to B and T cells in jawed vertebrates, expressing immunoglobulin receptors, known as variable lymphocyte receptors, instead of the immunoglobulin superfamily (34). These lymphocytes do not express RAG1/2 but instead express two cytidine deaminases, which replace the rearrangement function of RAG1/2 (35). In addition, lampreys harbor neither a thymus nor bone marrow, but rather a thymus counterpart named the thymoid (36). Thus, GALT in lamprey, termed the typhlosole, plays roles in hematopoiesis as well as lymphopoiesis. The site of the typhlosole depends on the life cycle stage since the body plan changes drastically as a result of developmental processes including metamorphosis (37).

Among jawed vertebrates, cartilaginous fish such as sharks is the oldest class which diverged from the other jawed vertebrates 450 million years ago (38). However, they already possess an acquired immune system with many basic cellular and molecular similarities to those of mammals. The spleen has already been described as a SLO in sharks, and it possesses organized lymphoid tissue, albeit no germinal center (39). Sharks have aggregates of GALT in spiral valves, which are structures that increase the intestinal wall surface area due to their extra folds and twists (40, 41). This structure is similar to, and considered a primitive ancestor of, Peyer’s patch in mammals. Nonetheless, M cells are not found in the epithelium of spiral valves (42, 43).

Bony fish appeared 400 million years ago (44). MALT in bony fish is composed of diffuse lymphoid aggregates without germinal centers (45) and is subdivided into six subtypes according to anatomical site: GALT, gill-associated lymphoid tissue, NALT, buccal MALT, pharyngeal MALT, and SALT (skin) (46). The presence of SALT was confirmed in a study of parasite infection in teleosts (47), in which a GALT-like structure was seen in the cutaneous mucosa, and B cells produced a polymeric form of a secretory immunoglobulin, IgT. SALT in bony fish was subsequently revealed to protect the organism from infection during the developmental period when GALT is nonfunctional (47). Although the presence of SALT is still disputed in humans, SALT functions in cutaneous local immunity in bony fish. Recently, a putative ancestral counterpart of mammalian M cells was discovered in salmonid intestines (48); however, it is unknown whether a functional equivalent of M cells exists in bony fish (49).

Lungfish are air-breathing sarcopterygian fish species that are considered the species closest to tetrapods (50), whose precursor appeared 420 million years ago (51). A recent study revealed that African lungfish harbors structured lymphoid follicles, the MALT structure in NALT of the upper and lower jaws. Furthermore, the epithelium above the MALT structure possesses M cells (52). Thus, African lungfish is considered the first organism in evolution to have an established architecture of the inductive site, although it lacks germinal centers (52).

It is assumed that divergence of mammal and amphibians occurred 350 million years ago (53). The spleen, liver, and kidney are the hematopoietic organs of the aquatic larval stage (tadpole). The spleen also serves as an SLO, although it lacks germinal centers. Bone marrow for B- cell production was firstly evolved in adult frogs. Furthermore, the presence of class-switched immunoglobulin corresponding to human IgG is phylogenetically the first in the adult frogs (54). Tadpole skin consists of a two-cell layer of the epidermis, which is covered by a mucus layer. In contrast, after metamorphosis, a 5–7-cell layer of the mucus-covered epidermis, which is covered by a monolayer of keratinized cells, develops in adult frog skin (55). Amphibians such as frogs harbor MALT and M cells, but the latter were observed only in the non-keratinized epithelium. Thus, whether tadpoles possess SALT similar to bony fish, despite a lack of SALT in adult frogs, is of great interest. However, no studies have addressed this question.

Birds and mammals are endotherms (warm-blooded animals). Birds evolved from theropod dinosaurs during the Jurassic approximately 165–150 million years ago (56). Though mammals were considered to be diverged from therapsids approximately150 million years, it remains to be concluded when the definitive mammal appeared (57). These organisms harbor the thymus, bone marrow, spleen, and MALT, although the presence of SALT has long been disputed (13). Notably, novel encapsulated lymphoid organs evolved among these organisms as SLOs. Lymph nodes are an SLO present only in mammals, while the bursa of Fabricius in birds which is considered the avian counterpart of the lymph nodes (9).

We can assume that MALT and its subtypes play a role in acquired immunity before the evolution of encapsulated organs dedicated to antibody production, such as the lymph nodes. However, functional analyses in this field are insufficient, and much of the existing evidence is descriptive only. The following intriguing evolutionary questions regarding the SALT concept postulated by Streilien have been raised. Does SALT (or iSALT) exist in aquatic organisms other than bony fish, such as sharks and tadpoles? Does SALT (or iSALT) share structures analogous to other TLSs? Finally, does MALT contribute to antibody production more than does the spleen among lower vertebrates? Answers to these question will clarify how universal the presence and functions of SALT are in vertebrates.

## FDCs and the Melanomacrophage Center (MMC)

In evolutionary history, the germinal center is absent in ectotherms (i.e., cold-blooded animals), first appearing in endotherms, corresponding to the evolution of FDCs (58). FDC serves as long-term storage of intact antigens and facilitates antigen–antibody interactions (59). It is questionable whether lymphoid follicles without germinal centers can perform affinity maturation or class switch recombination in antibody production. Lymphoid follicles without germinal centers are termed primary follicles, which are considered to be quiescent and composed mainly of naïve B cells that recognize cognate antigens (60). In contrast, lymphoid follicles with germinal centers are termed secondary follicles in which activated B cells undergo antibody affinity maturation (61). However, in a recent immunization study, affinity maturation of the selected antibody was seen, even in ectotherms (sharks), albeit with a relatively lesser degree of affinity increase compared to so-called higher vertebrates (62).

Recent studies have provided new evidence of a distinct type of pigment-containing cells, called melanomacrophages. Notably, melanomacrophages form aggregates in lymphoid follicles, which is called the MMC. Although the MMC is not detected in lamprey, a wide range of ectotherms, from hagfish among the jawless fish to almost all jawed animals (e.g., reptiles) harbor MMC (63). Nonetheless, it is not clear whether melanomacrophages are present in MALT. Intriguingly, melanomacrophages react with CNA.42 antibody which detects mammalian FDCs (64), and cells positive for activation-induced cytidine deaminase are positioned in proximity to melanomacrophages (65). Thus, it has been hypothesized that the MMC is a precursor of FDCs (63). However, functional analyses are still scarce, and the above mentioned research findings are descriptive and highly speculative.

However, these findings pertaining to the MMC raise the question of whether MALT and its subtypes are involved in acquired immunity, including in ectotherms, since MMCs have been detected mostly in the spleen, liver, and kidney (66). One study of shorthorn sculpins revealed a high frequency of MMCs in the spleen and liver, while only 3% of examined samples contained MMCs in the gill, which harbors gill-associated lymphoid tissue (67). Another study of gilthead seabream showed that MMC formation in the intestine was induced after feeding a diet of microalgae (Navicula) or a normal daily diet, although there was no mention of whether the MMCs were located in GALT (68). Furthermore, melanomacrophages were induced in the heart of goldfish (Carassius auratus) after chronic inflammation (69). Thus, some researchers speculate that MMCs can be induced by certain stimuli such as chronic inflammation, similar to iSALT formation under chronic inflammation. Thus, it might be possible that the MMC is a TLS in ectotherms.

Another intriguing aspect is that only endotherms form lymph nodes, Peyer’s patches, and germinal centers containing FDCs, all of which require lymphotoxin for development (11). This led us to further speculate that MALT, capsule-free lymphoid tissue, is a precursor of all SLOs, which encompasses MMC formation under certain stimuli such as inflammation. Lymph nodes might have evolved in endotherms mediated by the genetic program relating to lymphotoxin, which led to the evolutional substitution from MMC to FDC and encapsulation of the lymphoid organ dedicated to antibody production. This evolution might be triggered by the overwhelming demand for antibody production.

## iSALT Formation in a Mouse Model of CHS

For dermatologists and dermatopathologists, perivascular infiltration of lymphocytes is frequently encountered in daily practice. However, little is known about the pathophysiological mechanisms. While exploring the contribution of antigen-presenting cells (APCs) to perivascular infiltration in a mouse model of CHS, our group discovered a skin immune complex vital for T-cell activation. Based on the finding that it plays a role in local immunity, we termed this complex iSALT (70).

Cutaneous APCs are classified into two subsets, epidermal Langerhans cells (LCs) and dermal DCs (dDCs). First, we evaluated the antigen-presenting capacity of APC subsets in the skin. To deplete each cutaneous DC subset separately, we used transgenic mice expressing the gene encoding the diphtheria toxin receptor under the control of the langerin and CD11c promotors, in combination with the bone-marrow chimera technique. We depleted each APC subset by administering diphtheria toxin just before eliciting the CHS response and found that the ear swelling and inflammatory histological findings were significantly attenuated in the absence of dermal DCs but not LCs. Furthermore, the production of interferon-γ from skin-infiltrated T cells was suppressed in mice lacking dermal DCs. These results suggest that dermal DCs are essential for T cell activation and elicitation of CHS responses. Contact dermatitis in humans manifests as spongiosis, intercellular edema in the epidermis, and co-localization of perivascular infiltrates of T cells and DCs in the dermis, especially beneath the vesicles in the epidermis, suggesting that focal contact between T cells and DCs in the dermis might be essential to induce dermatitis.

Next, the dynamics of dermal DCs and skin-infiltrated T cells during the elicitation phase of CHS were analyzed *in vivo* in mice by multi-photon microscopy. In the steady state, dermal DCs showed diffuse distribution and random migration in the dermis. Upon antigen application, the dermal DCs transiently increased their migration velocity, formed clusters in the perivascular area, and interacted with T cells for several hours, suggesting that these DC/T cell clusters are antigen presentation sites in the skin. Further, we assessed whether T cells increase by the formation of DC/T cell clusters in the dermis. At 24 hours after CHS induction, the number of CD8+ T cells had increased in an antigen-dependent manner, whereas CD4+ T cells showed a low proliferative capacity. These findings indicated that the formation of DC/T cell clusters within the dermis in response to antigen induces proliferation and activation of CD8+ T cells in inflamed skin.

We next examined the factors that trigger DC/T cell cluster formation. We evaluated the number of DC clusters formed in a CHS model using mice that lack various immune cells and found that DC clustering was abrogated in mice depleted of macrophages and neutrophils by diphtheria toxin treatment. Depletion of both macrophages and neutrophils reduced ear swelling and production of interferon-γ by T cells. On the other hand, depletion of neutrophils alone did not interfere with the formation of DC clusters.

Next, the migratory kinetics of dermal macrophages and DCs *in vivo* was investigated by two photon microscopy. In a mouse model of hapten-induced CHS, dDCs were accumulated mainly around blood vessels. Time-lapse live imaging demonstrated that dDCs migrated toward perivascular macrophages. The results suggest that the formation of DC/T cell clusters plays a crucial role in T cell activation and requires dermal perivascular macrophages. Our subsequent analysis showed that keratinocyte-derived IL-1a activated perivascular macrophages during the elicitation phase, and that perivascular M2 macrophages were essential for DC cluster formation *via* CXCL2 production. Collectively, we conclude that perivascular clusters comprising DCs and M2 macrophages are essential to activate CD8+ T cells in the inflamed skin of a CHS mouse model, which we termed iSALT.

If iSALT functions in the same manner as MALT does, we speculate that the findings of this study might describe mechanistic insight of the effector site of iSALT (**Figure 1**). Thus, it is assumed that memory T cells circulate within a limited area of the skin, and that they accumulate before activation by local acquired immune stimuli. Furthermore, recent studies indicated that Langerhans cells in the epidermis function as a regulator of not sensitization phage but excitation phase. Thus, it is of great interest to know the mechanistic insight whereby inflammation of effector site is modulated by Langerhans cells which exclusively existed in the epidermis (71).

## iSALT in Humans

Dermatology textbooks describe various diseases involving co-localization of lymphoid follicles and plasma cells, raising the possibility that these entities are associated with transient induction of TLSs in the skin. Chronic inflammation is the key factor inducing TLSs; thus, we attempted to identify iSALT in chronic inflammatory skin disorders characterized by plasma cell infiltration.

**Figure 3:**
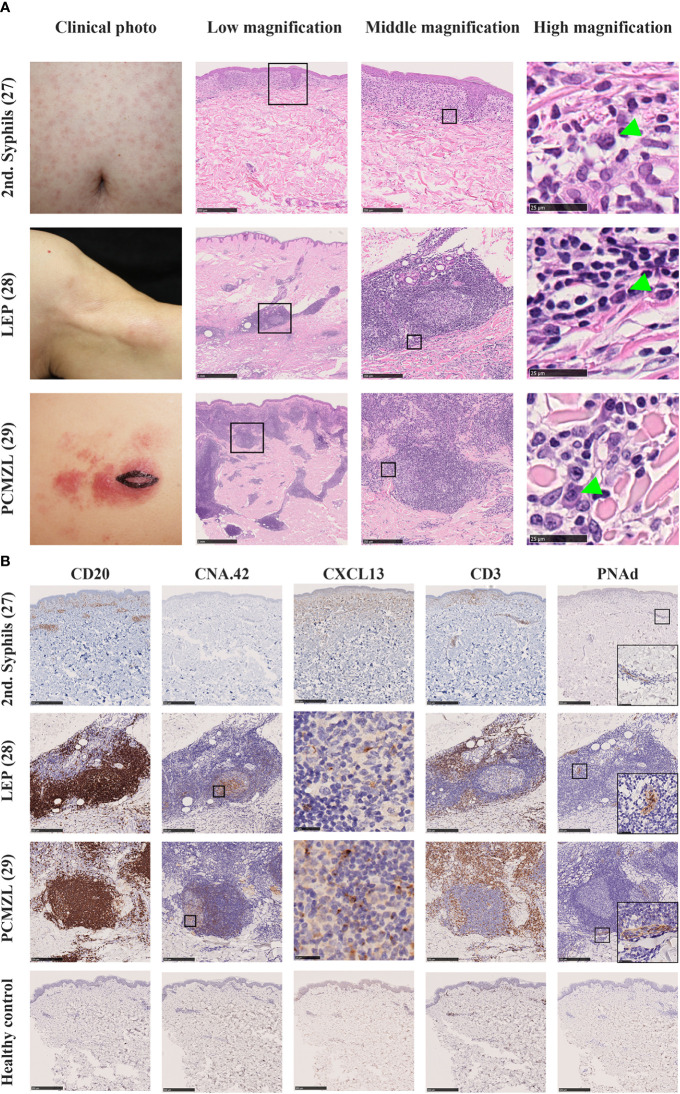
**(A)** Clinical pictures and H.E. staining of various human diseases which contain iSALT-like structures. Secondary syphilis (2^nd^ syphilis, Top Row) (26), lupus erythematosus profundus (LEP, Middle Row) (27), and primary cutaneous marginal zone cell lymphoma (PCMZL, Bottom Row) (28) are shown. In low magnification views of H.E. staining, lymphoid aggregates are seen in the dermis (scale bars: 500 μm in 2^nd^ syphilis and 1 mm in the others). In middle magnification views from the boxed areas in the low magnification, lymphoplasmacytic infiltration is seen in 2^nd^ syphilis while iSALT-like structures are formed in LEP and PCMZL lesions (scale bar: 250μm). In high magnification views from the boxed areas of middle magnification views, we can see plasma cells (green arrowheads, scale bar: 25μm). **(B)** Immunohistochemistry for components of TLS in iSALTs. Lymphoid aggregates in 2^nd^ syphilis and lymphoid follicles in LEP and PCMZL were stained with antibodies of anti-CD20 (to detect B cells), anti-CXCL13 (to detect chemo-attractant for B cells), CNA.42 (to detect FDC), anti-CD3 (to detect T cells), and anti-PNAd (to detect high endothelial venules). Scale bars: 500 μm in 2^nd^ syphilis and 250 μm in LEP and PCMZL (except for CXCL13). CXCL13 stainings in LEP and PCMZL were from the boxed areas of CNA.42 stainings. Positive signals were seen in the germinal centers (scale bars: 25 μm). PNAd staining includes the magnified views of the boxed areas in the right bottom corners. Healthy control were also stained with the same antibody panel (Bottom low, scale bar: 500 μm).

Secondary syphilis manifests as maculopapular eruptions due to chronic infection with spirochetes, *Treponema pallidum*. The histopathological diagnosis of this disease is based on plasma cell infiltration in the lesional skin. In addition, enrichment of CXCL13 in the cerebrospinal fluid of neurosyphilis patients has been reported (72). Thus, we speculated that plasma cells in the lesions might be a sign of the presence of skin TLSs that induce local differentiation of the B-cell lineage (**Figure 3A**, Top Row). Immunohistochemical analysis revealed the presence of diffuse B- and T-cell aggregations in the dermis but no lymphoid follicles; however, CXCL13 was diffusely expressed in fibroblast-like cells in the superficial dermis, and Peripheral lymph node addressin (PNAd)+ vessels that differentiate into HEVs were distributed sparsely in the dermis (**Figure 3B**, Top Row). Thus, we speculate that these are disassembled components of TLSs in human skin, which are the human counterpart of iSALT (26).

Next, we assessed the skin lesions of another chronic inflammatory disease, lupus erythematosus profundus (LEP) (27). LEP is characterized by the presence of lymphoid follicles with plasma cell differentiation. Furthermore, approximately 45% of LEP cases display germinal centers within the lesion (73). In our histological analysis of LEP, hematoxylin and eosin staining showed that inflammatory cells were densely infiltrated in the dermis and formed multiple lymphoid follicles (**Figures 3A, B**, Middle Row). These lymphoid follicles are composed of a CD20+ B-cell zone, CXCL13+ CNA.42+ FDCs, and CD3+ T cells surrounding the B-cell zone. PNAd+ HEVs were observed in the T-cell zone in all samples. Thus, the follicles displayed a similar immunohistochemical pattern to that of TLSs (**Figure 3B**, Middle Row).

Although TLSs including iSALT are considered to be induced by inflammation, iSALT formation can be observed in the lesional skin of patients with primary cutaneous marginal zone lymphoma (PCMZL). PCMZL was previously called cutaneous MALT lymphoma since it tends to be indolent and differentiates into tumor cells recapitulating the cells of Peyer’s patch (74). PCMZL is subdivided into two subtypes: class-switched and non-class-switched. The class-switched subtype manifests as well-circumscribed lymphoid follicles containing plasma cells expressing class-switched immunoglobulins such as IgA or IgG. The non-class-switched subtype is characterized by diffuse infiltration of IgM-expressing B cells. Notably, the class-switched subtype was associated with a much better prognosis (5-year survival rate, 99–100%) compared with the non-class-switched subtype (75, 76). Hence, the class-switched subtype is assumed to be an inflammatory entity rather than an overt lymphoma. Intriguingly, PCMZL is hypothesized to derive from the putative TLS in the skin, that is, SALT. Therefore, some proposed the term PCMZL as SALToma (77). We assessed whether PCMZL encompassed the characteristics of iSALT (28). Hematoxylin and eosin staining of PCMZL exhibited dense infiltration of inflammatory cells along the skin appendages, which is described as a “blue column in the dermis”. Furthermore, lymphoid follicles were scattered among the inflammatory aggregates (**Figure 3A**, Bottom Row). Briefly, CD20+ B-cell zones contained FDCs overlapping the sites of CXCL13 expression, and T-cell zones were composed of CD3+ T cells interspersed with PNAd+ HEVs (**Figure 3B**, Bottom Row), whose immunohistochemical pattern was analogous to that of LEP (**Figure 3**, Middle Row). Thus, we concluded that PCMZL harbors the characteristics of iSALT. This suggests the important notion that the lymphocytes and presumably B-cell lymphoma cells that develop in iSALT migrate predominantly within the skin. Thus, the clinical manifestation of PCMZL, which involves no organ other than the skin, can be well-explained by the SALT concept postulated by Streilein.

Collectively, we confirmed that various skin diseases are associated with iSALT. Nonetheless, current knowledge about iSALT is no more than TLS in histology-based study. Thus, it is essential to clarify mechanistic insights of iSALT to comprehend the local immunity of the skin.

## Summary and Perspectives

In this review, we summarized the concept of MALT, the evolution of lymphoid tissue among vertebrates, and the current knowledge on iSALT. From an evolutionary point of view, MALT exists among all vertebrates, although the architecture of MALT has evolved over time. It is speculated that this non-encapsulated lymphoid tissue has a long history of regulating vertebrate immunity. Furthermore, conservation of MALT implies the possibility that genetic and cellular component of MALT might serve as a primordium for many SLOs. Thus, the spleen might emerge from GALT with encapsulation as an independent organ in jawed animals which plays roles in hematopoiesis and red blood cell clearance as well (78). Subsequently, lymph nodes and its counterparts might evolve as additional organs dedicated to systemic function of acquired immunity in mammals and birds. Nonetheless, this research field is still supported mainly by descriptive studies, and the pieces of evidence validated by functional experiments are still scarce.

However, the knowledge about MALT and TLSs provide a wide field of vision to foresee the whole picture of the biological significance of iSALT. Thus, we can recapitulate the following biological questions:

1. Are lymphoid follicles in various tissue (including ectopic ones, reactive ones or any other lymphoid neogenesis) same as TLSs?
2. Do TLSs including iSALT have the same architecture as that of MALT, supported by similar gene expression?
3. Do TLSs including iSALT harbor local circulating lymphocytes, which belong to each TLS?
4. Do TLSs including iSALT encompass the same immunological functionality?

These questions will answer whether all TLSs defined by histology-based study, including iSALT, contributes to the local immunity in contrast with lymph nodes that play pivotal roles in systemic immunity. Thus, this research would be able to re-introduce the notion of SALT which Streilein postulated into various skin diseases that frequently accompanies with lymphoid follicle formation such as pseudolymphoma, primary cutaneous plasmacytosis, Kimura disease, and so on. We believe that the answer to this question is essential to comprehend the whole picture of local immunity including the skin immunity.

## Author Contributions

TK contributed to the conceptualization of inducible skin-associated lymphoid tissue (iSALT) and writing the first draft of a manuscript. KK contributed to the conceptualization of iSALT, and revising the manuscript. GE contributed to revising the manuscript. All authors contributed to the article and approved the submitted version.

## Funding

Takeda Sciences Foundation, Grants-in-aid for Scientific Research (20K08649 and 21K16227).

## Conflict of Interest

The authors declare that the research was conducted in the absence of any commercial or financial relationships that could be construed as a potential conflict of interest.

## Publisher’s Note

All claims expressed in this article are solely those of the authors and do not necessarily represent those of their affiliated organizations, or those of the publisher, the editors and the reviewers. Any product that may be evaluated in this article, or claim that may be made by its manufacturer, is not guaranteed or endorsed by the publisher.
